# Optical Penetration of Shape-Controlled Metallic Nanosensors across Membrane Barriers

**DOI:** 10.3390/s23052824

**Published:** 2023-03-04

**Authors:** Ancheng Da, Yanan Chu, Jacob Krach, Yunbo Liu, Younggeun Park, Somin Eunice Lee

**Affiliations:** 1 Department of Electrical & Computer Engineering, Biomedical Engineering, Biointerfaces Institute, Applied Physics, Macromolecular Science & Engineering, University of Michigan, Ann Arbor, MI 48109, USA; 2 Department of Mechanical Engineering, University of Michigan, Ann Arbor, MI 48109, USA

**Keywords:** optical force, photothermal, plasmonics, bioplasmonics

## Abstract

Precise nanostructure geometry that enables the optical biomolecular delivery of nanosensors to the living intracellular environment is highly desirable for precision biological and clinical therapies. However, the optical delivery through membrane barriers utilizing nanosensors remains difficult due to a lack of design guidelines to avoid inherent conflict between optical force and photothermal heat generation in metallic nanosensors during the process. Here, we present a numerical study reporting significantly enhanced optical penetration of nanosensors by engineering nanostructure geometry with minimized photothermal heating generation for penetrating across membrane barriers. We show that by varying the nanosensor geometry, penetration depths can be maximized while heat generated during the penetration process can be minimized. We demonstrate the effect of lateral stress induced by an angularly rotating nanosensor on a membrane barrier by theoretical analysis. Furthermore, we show that by varying the nanosensor geometry, maximized local stress fields at the nanoparticle–membrane interface enhanced the optical penetration process by four-fold. Owing to the high efficiency and stability, we anticipate that precise optical penetration of nanosensors to specific intracellular locations will be beneficial for biological and therapeutic applications.

## 1. Introduction

Precision genome engineering ultimately relies on delivery vehicles carrying editing components to be efficiently delivered across cell membranes [1]. Furthermore, direct and precise delivery to a specific intracellular location would be beneficial to ensure high efficacy of gene editing. Real-time observation over prolonged time periods is needed to visualize the entire delivery and gene editing processes, but this information is currently lacking. Nonbleaching nanosensors as multifunctional optical readout and delivery vehicles hold promise for enabling a new way to sense gene editing in real time [2,3,4]. The cell plasma membrane serves as a barrier to prevent large macromolecules from crossing into the intracellular environment. Since the cell plasma membrane forms a stable impenetrable barrier, delivery of macromolecules, such as gene editing components, across membrane barriers remains a significant obstacle [1]. To overcome this problem, various viral and non-viral methods have been developed for delivery across membrane barriers [5]. Viral methods [6,7] employ viruses to inject gene editing components across the cell membrane. Non-viral methods [8,9,10], such as lipofection and electroporation, have been used to chemically and/or physically disrupt the cell membrane to deliver gene editing components. However, to date, achieving precise and direct delivery to desired intracellular locations remains difficult.

Recently, optical forces [11,12,13,14,15,16,17,18,19,20,21] enabled precise manipulation of nano-objects [22,23]. The optical force on nano-objects consists of two major contributions, the scattering force and the gradient force. The gradient force acts to attract the nano-object towards the focus of the light beam while the scattering force acts to repel the nano-object along the light propagation direction. Using a tightly focused Gaussian laser beam, forward-directed scattering force has been utilized to inject metallic nano-objects, such as gold nanoparticles, through membranes. However, in metallic nano-objects [24,25,26], a third contribution from absorption becomes significant. For instance, in the case of gold nanoparticles, due to their strong and sharp resonance peak wavelength in their optical extinction properties, gold nanoparticles efficiently absorb and convert light energy into photothermal heat. This photothermally generated heat can be potentially damaging to cells [27]. While optical trapping of gold nanoparticles can be achieved away from the resonance wavelength, photothermal heat generation is still challenging.

Photothermal heat generation can be mitigated by separating the gradient force from the scattering force and absorption. For this purpose, a radially polarized laser beam has been proposed to trap gold nanoparticles [17], although not in the context of membrane penetration. In radial polarization, separation of the gradient force from the scattering force and the absorption is achieved due to the scattering force and the absorption along the optical axis being zero while the non-propagating axial component contributes to the gradient force. Furthermore, superposition of a radially and azimuthally polarized beam can allow for vertical orientation of a nano-object [28]. Such a donut-shaped vector beam has an additional benefit as the optical intensity near the center where the particle is trapped is relatively low, which further decreases the severity of photothermal heat generation. While angular rotation of horizontally oriented nano-objects has been demonstrated [13,18], to the best of our knowledge, angular rotation of vertically oriented nano-objects has not been reported.

**Figure 1:**
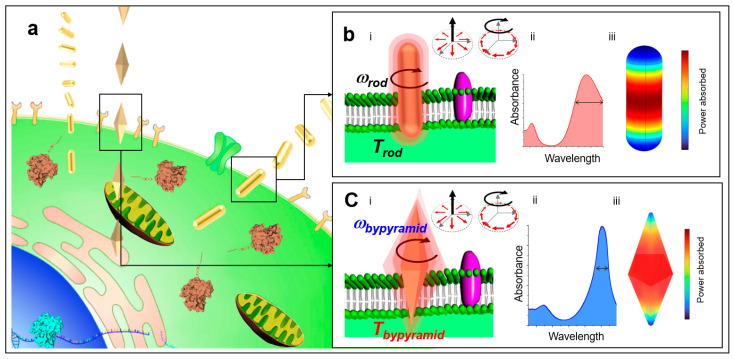
**Optical penetration of shape-controlled metallic nanosensors across cell membrane barriers.** (**a**) Conceptual schematic of nanosensors carrying editing components to be delivered across cell membrane barriers. (**b**) Conceptual schematic of AuNR nanosensor delivery: (**i**) penetration across cell membrane barrier, (**ii**) absorbance spectrum: absorption cross-section versus wavelength, (**iii**) three-dimensional model of power absorbed for AuNR nanosensor. (**c**) Conceptual schematic of AuBP nanosensor delivery: (**i**) penetration across cell membrane barrier, (**ii**) absorbance spectrum: absorption cross-section versus wavelength, (**iii**) three-dimensional model of power absorbed for AuBP nanosensor.

In this work, we present a new principle of angularly rotating vertically oriented metallic nanosensors for enhancing membrane penetration (Figure 1). Unlike conventional horizontally oriented metal nanosensors [11,13], angularly rotating vertically oriented metallic nanosensors can substantially enhance the applied force normal to the membrane but has not been yet demonstrated. Furthermore, engineering precise nanostructure geometry [29,30,31] with sharp edges and gradually varying cross-sectional areas can be exploited to pierce or drill through membrane barriers. In essence, we propose vertically oriented nanosensors as multifunctional nanodrills utilizing optical rotational forces. We show that by altering the nanostructural geometry of the nanosensor, we maximize optical penetration depth while minimizing photothermal heat generated during optical penetration. We develop a theoretical model of lateral stress induced by an angularly rotating, vertically oriented nanosensor on a membrane barrier. Furthermore, we show that by adjusting the nanosensor geometry, we effectively increase local stress fields at the nanoparticle–membrane interface, thereby enhancing the optical penetration process. Direct and precise delivery of nanosensors to specific intracellular location opens a wide range of biological and therapeutic applications [2,11,32,33,34,35,36,37,38,39,40,41,42,43,44,45], ranging from precision genome engineering to real-time ultraprecision imaging to quantitative medicine.

## 2. Results and Discussion

**Figure 2:**
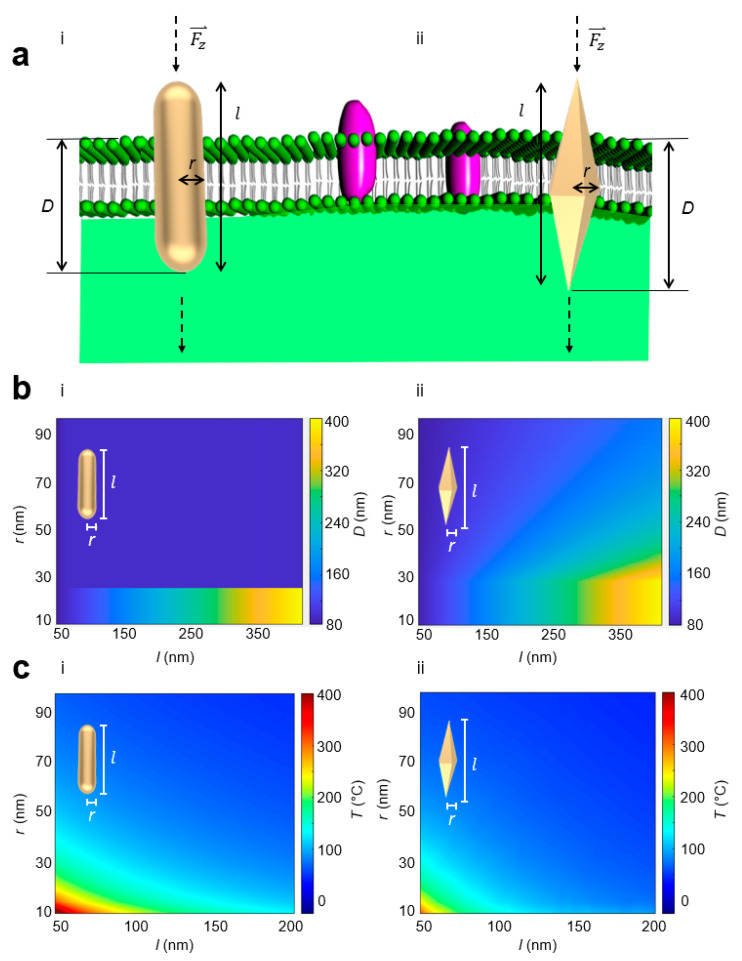
Penetration depth by applied normal force is greater for the AuBP nanosensor compared to the AuNR nanosensor. (**a**) Conceptual schematic of optical penetration of (**i**) AuNR nanosensor and (**ii**) AuBP nanosensor. ${\vec{F}}_{z}$: normal force, l: total length of nanoparticle, *r*: radius at the center of nanoparticle, *D* = optical penetration depth defined as the depth at which normal stress on the membrane reached below the critical *σ_z_*. (**b**) Comparison of optical penetration depth *D* as a function of geometric parameters *r* and l for (**i**) AuNR nanosensor and (**ii**) AuBP nanosensor. (**c**) Comparison of maximum local temperature when excited with *P* = 45 μW laser estimated based on FEA as a function of geometric parameters *r* and l for (**i**) AuNR nanosensor and (**ii**) AuBP nanosensor.

Superposition of a radially and azimuthally polarized vectorial beam can allow for three-dimensional (3-D) orientation of a nano-object [28]. The vectorial beam polarization interacts with nano-objects aligned parallel with the polarization direction. When the single vectorial beam is tightly focused through a high numerical aperture objective, it is possible for the electric field to have a significant longitudinal component as a result of destructive interference of the transverse components [46]. In this way, nano-objects can be vertically oriented. Light can exert forces on matter by transferring angular momentum from light to the nano-object. Spin angular momentum at the focal plane from the orbital-to-spin angular momentum conversion [47] can be then utilized to rotate vertically oriented nano-objects in order to rupture membrane barriers. Furthermore, focused higher order vector beams [48] that simultaneously contain radial, azimuthal, and longitudinal components where the relative magnitudes of these components can be tuned to achieve flexible and complex nano-object orientation and rotation. The strong longitudinal component aligns the nano-object vertically, while the radial component optically induces rotation. In contrast to previously demonstrated horizontally oriented nano-objects that rotate about their short axes normal to the optical axis [11,13], we studied the effect of nanostructural geometry of vertically oriented nano-objects using two distinct metallic nanosensor geometries: gold nanorod (AuNR) nanosensors and gold bipyramids (AuBP) nanosensor. AuBP nanosensors have a pentagonal cross-section, and the AuNR nanosensors possess a circular cross-section. The AuBP nanosensors display a narrower spectral bandwidth compared to the AuNR nanosensors, which is beneficial for optical trapping (Figure 1). Membrane rupture is ultimately required for a delivery nanosensor to reach an intracellular site. In the vertical orientation, we conjectured that the variable surface area along the length of the AuBP nanosensors may enhance the penetration process. We reasoned that when vertically oriented, the radius of the AuBP nanosensors gradually increases at a slower, linear pace until the maximum radius at the center is reached as compared to the AuNR nanosensors in order to aid the penetration process. This results in the AuBP nanosensor achieving a deeper penetration before reaching the same cross-sectional area as compared to the AuNR nanosensor. The geometry is defined by length *l* and radius *r* measured at the center of the nanosensor shown in Figure 2. We assumed the membrane sustained a constant force ${\vec{F}}_{z}$ in the direction normal to the membrane, creating normal stress on the membrane. The normal stress is defined as *σ_z_* = $\vec{\left|F_{z}\right|}$/A, where $\vec{F_{z}}$ is the applied force normal to the membrane and *A* is the cross-sectional area of the nanosensor. At a critical *σ_z_*, defined as the membrane rupture critical threshold *σ_c_*, the membrane will rupture, allowing the nanosensor to penetrate through the membrane barrier. Previous research in the literature [49] measured *σ_c_* to be 10 kPa. Figure 2 depicts the penetration depth, *D*, defined as the depth at which normal stress on the membrane reaches below the critical *σ_z_* (i.e., length of the nanosensor passing through the membrane barrier). *D* was calculated while iterating through the range of combinations of geometrical parameters *r* and *l* to yield Figure 2. For a rod-shaped geometry, *D* equaled the length of the nanosensor (i.e., full penetration) when the radius was below 25 nm, but immensely fell off when the radius exceeded 25 nm. This is explained by the failure of the nanosensor to penetrate the membrane barrier as the cross-sectional area of the AuNR nanosensor quickly became large. Horizontally oriented nano-objects also suffer from this limitation, as the cross-sectional area interfacing the membrane is significantly large compared to angularly rotating vertically oriented nano-objects. Hence, it remains challenging to utilize horizontally oriented nano-objects to penetrate through membrane barriers. In comparison, the AuBP nanosensor displays similar behavior at radii under 25 nm, but provides a much more dynamic response at radii greater than 25 nm, attributed to the increased efficacy of penetration with more sharp edges and more gradual cross-sectional area changes.

Metallic nano-objects efficiently absorb and convert light energy into photothermal heat, which can be potentially detrimental to biological cells. Thus, we then investigated whether photothermal heat generation can be minimized by varying nanostructure geometry using finite element analysis (FEA). We compared the generation of photothermal heat in the AuNR nanosensors and the AuBP nanosensors. In this investigation, we assumed the complex permittivity to be a function of wavelength [50] and the relative permeability of gold *μ_r_* = 1. Over a range of geometrical parameter *r* from 5 to 200 nm and a range of geometrical parameter *l* from 50 to 400 nm, the nanosensor rested within an aqueous exterior and was excited with a tightly focused single vectorial beam with a laser power of 45 μW in the near-infrared wavelength regime (700 nm–900 nm) [23]. The membrane was assumed to be fluid with permeability of *μ_r_* = 1 and permittivity between 2–10 [51]. Since the nanosensor was in contact with the membrane, it can be reasonably assessed that the temperature of the membrane is close to the temperature of the nanosensor at the nanoparticle–membrane interface. The resulting surface temperatures of both the AuNR nanosensor and the AuBP nanosensor at specific geometrical parameters *r* and *l* can be observed in Figure 2. At similar geometrical parameters *r* and *l*, we found a lower maximum temperature for the AuBP nanosensors, indicated by a peak temperature of 412 °C for the AuNR nanosensor against 340 °C for the AuBP nanosensor at *r* = 10 nm and *l* = 50 nm. Taken together, these results show that penetration depth can be maximized while photothermal heat generated can be minimized by varying the nanosensor geometry. Minimization of photothermal heat generation during optical penetration addresses one of the current limitations in nanosensor-delivery-based methods. Furthermore, to decouple photothermal heat generation from optical penetration, we considered a dielectric material (silica nanorod (siNR) and silica bipyramid (siBP)), which do not undergo the same level of photothermal heat generation in Figure S1. At all input laser levels, gold nanosensors underwent more photothermal heat generation than silica nanosensors, alongside the expected increase in heat generation of the nanorod compared to the bipyramid. To further distinguish nanostructural geometry (rod vs. bipyramid) from mechanical property changes induced by photothermal heat generation, we compared the lateral stress generated by the nanosensors at variable temperatures. Figure S2 shows the gradual decrease in lateral stress as temperature increases for both the nanorod and the bipyramid, as viscosity of the membrane is affected. The bipyramid nanosensor outperformed its respective nanorod counterpart in generating lateral stress at all temperatures. These results show that even though temperature may have an effect on the relative lateral stress generated by the bipyramid and the rod, nanostructural geometry is still primarily responsible for the stress variation. In physiological conditions, bipyramidal nanosensors are preferable for optical penetration of membrane barriers.

**Figure 3:**
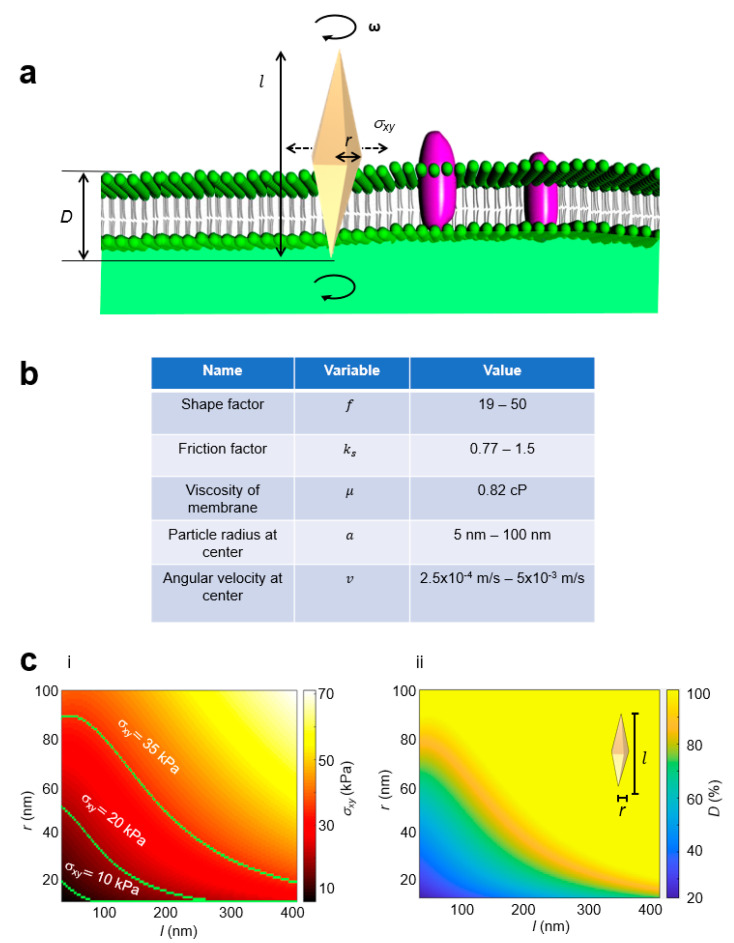
**Penetration with angular rotation generated lateral stress at the membrane.** (**a**) Conceptual schematic of the lateral stress model. *ω* = angular rotation, *σ_xy_* = lateral stress, l: total length of nanoparticle, *r*: radius at the center of nanoparticle. (**b**) Table of parameters for the calculation of *σ_xy_*: shape factor *f*, friction factor *k_s_*, viscosity of the membrane *μ*, particle radius at the center a, and angular velocity at the center v. (**c**) (**i**) Minimum lateral stress generation as a function of geometrical parameters *r* and l. (**ii**) Penetration depth *D* as a function of geometrical parameters with a membrane rupture critical threshold of *σ_c_* = 35 kPa. Penetration depth *D* is defined as the depth at which normal stress on the membrane reached below the critical *σ_z_*.

We next studied whether angularly rotating, vertically oriented nano-objects can further enhance membrane penetration (Figure 3). We proposed to orient a nanosensor in the vertical direction to maximize the normal force applied on the membrane. To this end, we developed a theoretical model of lateral stress induced by an angularly rotating nanoparticle on a membrane. Angular rotation rates *ω* ~50 kHz have been previously generated [13]. When a nanosensor is subject to an external rotating force while trapped on the membrane, the rotational friction force is balanced by the external rotating force. Assuming the membrane is fluid with a sufficiently low Reynolds number [52], the rotational friction force adapted from a spherically shaped nanosensor [53] to a bipyramidal shaped nanosensor can be written as

(1) $$\vec{F_{xy}}=S\mu av$$

where $S=fk_{s}\pi$ is the coefficient adapted for a bipyramidal nanosensor (see Methods), *f* is the shape factor [52,54], *k_s_* is the friction factor [55], *µ* is the viscosity of membrane [56], a is the radius of the nanosensor at the center [40,57,58], and v is the angular velocity of the nanosensor at the center [59]. The lateral stress is then defined as *σ_xy_* = $\vec{\left|F_{xy}\right|}$/*A*, where *A* is the surface area of the membrane interface. As the AuBP nanosensor is vertically oriented, this nanosensor has a variable surface area at the area where friction is generated, as each “slice” of nanosensor has different geometric properties. Thus, we considered the entire lateral stress generated by rotation to be applied to a small, thin area of particle that is currently penetrating into the membrane and discounted any lateral stresses generated by the surrounding fluid. In Figure 3, with an angular rotation rate of *ω* = 50 kHz, we plotted the results of Equation (1) with different nanosensor geometrical parameters. We observed that increasing either geometrical parameter *r* or *l* of the nanosensor increased the lateral stress generated (Figure 3c). Three curve lines corresponding to *σ_xy_* = 10, 20, and 35 kPa stress generation were plotted and can be referred to as threshold penetration curves, in which a membrane with the corresponding membrane rupture critical threshold *σ_c_* allowed a nanosensor above the penetration curve to penetrate fully, while a nanosensor underneath the curve only allowed for partial penetration. Figure 3c displays the full percentile penetration depths of nanosensors interacting with a membrane with a membrane rupture critical threshold *σ_c_* = 35 kPa. From this result, we observed that changing the geometrical parameters *r* and *l* of the nanosensor enhanced the percent penetration depths. Of note, changes in geometrical parameter *r* corresponded to larger changes in percent penetration depths compared to changes in geometrical parameter *l* of the nanosensor.

**Figure 4:**
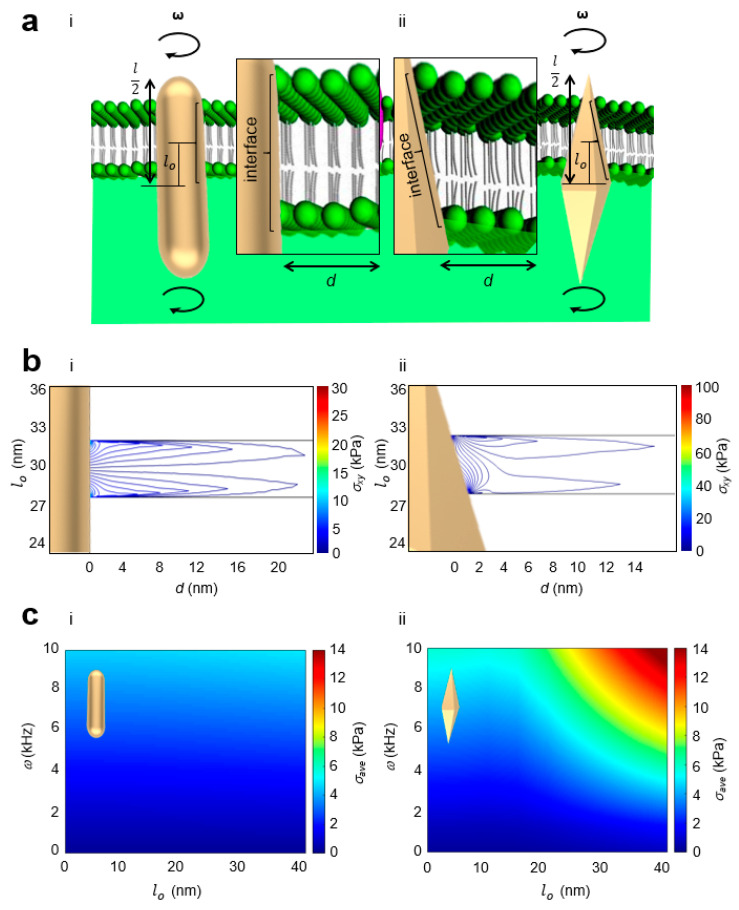
Local lateral stress at the nanoparticle–membrane interface increased penetration depth for the AuBP nanosensor compared to the AuNR nanosensor. (**a**) Conceptual schematic of average stress generation at the membrane interface and inset of membrane interface for (**i**) AuNR nanosensor and (**ii**) AuBP nanosensor. *ω* = angular rotation, l: total length of nanosensor, $l_{o}$: offset between center of nanosensor and center of membrane. (**b**) Local lateral stress contours at the nanoparticle–membrane interface for (**i**) AuNR nanosensor and (**ii**) AuBP nanosensor. $l_{o}$ = 30 nm, *ω* = 10 kHz. (**c**) (**i**) Average lateral stress at the membrane–nanoparticle interface as a function of $l_{o}$ and *ω* for the AuNR nanosensor. (**ii**) Average stress at the membrane–nanoparticle interface as a function of $l_{o}$ and *ω* for the AuBP nanosensor.

To better understand the spatial localization of lateral stress, we further performed theoretical studies of AuNR and AuBP nanosensors vertically oriented normal to the membrane (Figure 4). The nanosensor was modeled as a two-dimensional (2-D) axisymmetric geometry, and the fluid domains were rotated at the angular rotation rates *ω* between 500 Hz to 10 kHz [59] to simulate the rotational forces. Assuming the viscosity of the membrane to be 0.3 Pa·s [56], the resulting lateral stress at the interface between the membrane and nanosensor was simulated. At a fixed penetration depth, we defined the offset $l_{o}$ to be between the center of the nanosensor and the center of the membrane. We found that the lateral stress localized at the beginning and at the end of the interface (Figure 4b). For the AuNR nanosensor, the interface remained constant along the length of the nanosensor. Thus, the lateral stress generated remained constant as a function of $l_{o}$. In contrast, the interface was variable along the length of the AuBP nanosensor, resulting in a variable lateral stress generated as a function of $l_{o}$ (Figure S3). Notably, we observed higher lateral stress generated along the tips of the AuBP nanosensor, thereby enhancing membrane penetration. In Figure 4c, we computed the average lateral stress *σ_ave_* at the membrane–nanoparticle interface as a function of $l_{o}$ and *ω*. We observed a positive correlation between $l_{o}$ and *σ_ave_*, indicating that more stress was generated when the membrane surrounded the tips of the AuBP nanosensor due to the smaller cross-sectional area of the AuBP nanosensor. We observed a four-fold higher *σ_ave_* for the AuBP nanosensor compared to the AuNR nanosensor. Engineering the nanostructure geometry to enhance optical penetration can aid in the precise and specific biomolecular delivery of nanosensors.

In discussion, we demonstrated significantly enhanced optical penetration of metallic nanosensors by engineering nanostructure geometry with minimized photothermal heating generation for penetrating across membrane barriers. Metallic nanosensors in aqueous media carry electric charge. Metallic nanosensors can have widely varying surface chemistry and charge, which can affect optical penetration. In principle, it is possible that modifying the surface chemistry and charge could further enhance optical penetration in a synergistic manner. Varying the surface chemistry and charge has been shown to increase the electric polarizability, leading to enhancements in optical forces in the femtonewton range [60]. Enhancing optical penetration in the piconewton range has not yet been explored so far and presents a future opportunity of study in further maximizing optical penetration. This work can be also extended to other types of nanosensors exhibiting anisotropic geometries, such as dielectric nanosensors, in the future.

## 3. Conclusions

In summary, optical penetration exhibiting minimized photothermal heat generation aids delivery of nanosensors to the desired intracellular sites for biological and therapeutic applications. We introduced a new concept of angularly rotating vertically oriented metallic nanosensors for enhancing membrane penetration. We show that by varying the nanosensor geometry, optical penetration depth can be maximized while photothermal heat generated during optical penetration can be minimized. We derived a theoretical model of lateral stress induced by an angularly rotating, vertically oriented nanosensor on a membrane. We show that by varying the nanosensor geometry, local stress fields at the nanoparticle–membrane interface can be maximized to aid the optical penetration process. We show that optical penetration of nanosensors, serving as multifunctional nanodrills, exhibit minimized photothermal heat generation. We anticipate that such multifunctional nanosensors will lead to safer and more effective methods of penetrating, transporting, and detecting nanosensors across biological cell membranes in real time, which is beneficial for precision biological and therapeutic applications.

## 4. Methods

### 4.1. Normal Stress

MATLAB software was used to compute the normal stress generation and the penetration depth. Nanosensor radii ranged from 20 nm to 200 nm and nanosensor lengths ranged from 50 nm to 400 nm based on experimentally feasible geometries [40]. The radii corresponded to the maximum radius at the center of the nanosensor. First, cross-sectional area at multiple points along the length of the nanosensor was calculated for each radii/length pair, and then the input normal force ${\vec{F}}_{z}$ = 15 pN [23] was used to find the normal stress function. Given the critical stress (*σ_z_* = 10 kPa), the point at which the stress failed to exceed this value was reported as penetration depth. Penetration depths were calculated while iterating through the range of combinations of radii and lengths.

### 4.2. Photothermal Heat

COMSOL software ver. 5.6 was used to solve the heat transfer equation: $\rho C_{p}u\cdot \nabla T-k\nabla \cdot \nabla T=Q$ for both rod and bipyramidal nanosensors using the heat transfer COMSOL module. The nanosensor rested within an aqueous exterior, and was excited with a tightly focused single vectorial beam [28] of laser power of 45 μW in the near-infrared regime (700 nm–900 nm) [23]. With multiple geometric parameter sweeps of radii 5 nm–200 nm and lengths of 50 nm–400 nm, we used a constant aqueous exterior of 5 µm. We assumed the complex permittivity to be a function of wavelength [50] and the relative permeability of gold *μ_r_* = 1. The membrane was assumed to be fluid with permeability of *μ_r_* = 1 and permittivity between 2 F/m–10 F/m [51]. Triangle-type meshing was utilized with a minimum and maximum element size of 0.1 µm and 0.8 µm, respectively. The temperature of the outmost surface was set to be 293.15 K as the boundary condition. The temperature ranged from 40 °C at a 200 nm radius and 400 nm length to 240 °C at a 5 nm radius and 50 nm length. Four nanosensors (AuNR, AuBP, siNR, siBP) of nominal size (*r* = 50 nm, aspect ratio = 4) were exposed to laser powers between 10 μW to 100 μW. Absorbed power was based on absorption cross-section. Average temperature was measured along the interface between the nanosensors and the surrounding fluid environment.

### 4.3. Lateral Stress

COMSOL software ver. 5.6 was used to solve the incompressible Navier–Stokes (ρ∇·u=0) equations for the bipyramidal nanosensor using the laminar flow (spf) COMSOL module. The nanosensor was modeled as a 2D axisymmetric geometry, and the fluid domains were rotated at angular rotation rates *ω* between 500 Hz to 10 kHz [59] to simulate the rotational forces. The resulting stress at the interface between the membrane and the nanoparticle was calculated. The material properties and viscosity of this liquid exterior was set to a viscosity of 8.2 × 10^−4^ cP and the viscosity of the membrane was set to 0.3 Pa·s [56]. We assumed the complex permittivity to be a function of wavelength [50] and the relative permeability of gold *μ_r_* = 1. Triangle-type meshing was utilized with a minimum and maximum element size of 1 nm to 50 nm, respectively. The boundary conditions were set to be no-slip at the edges of the model (walls). Lateral stress was measured using rod and bipyramidal nanosensor models. The effect of temperature variation from 20 °C to 60 °C was expressed as a change in the viscosity of the membrane. A gradual decrease in viscosity from 0.3 to 0.04 Pa·s was applied as temperature was increased [61].

### 4.4. Shape Factor

For a bipyramidal nanosensor, the shape factor [52,54] was defined as

$$f=\left(\frac{1}{3}\right)\left(2\sqrt{\psi }+\sqrt{\Psi }\right)$$

where ψ is the surface area sphericity (ratio of the surface area of a sphere with identical volume as the nanosensor to the actual surface area of the nanosensor) and Ψ is the cross-sectional sphericity (ratio of the cross-sectional area of a sphere with identical volume as the nanosensor to the actual cross-sectional area of the nanosensor).

### 4.5. Friction Factor

Previous research in the literature [55] reported the effect of complex shaped particles and geometrical parameter aspect ratio on hydrodynamic properties in viscous media. Based on the previous literature [55], we plotted the friction factor *k_s_* as a function of geometrical parameter aspect ratio (Figure S4).

## Data Availability

Correspondence and requests for data should be addressed to S. E. L.

## Supplementary Materials

The following supporting information can be downloaded at: https://www.mdpi.com/article/10.3390/s23052824/s1, Figure S1: Photothermal heat generation of metallic-based and dielectric-based nanosensors; Figure S2: Lateral stress of AuNR and AuBP under environmental temperature variation; Figure S3: Local lateral stress at the nanoparticle-membrane interface; Figure S4: Friction factor.
