# Supplementary material for: Optical Penetration of Shape-Controlled Metallic Nanosensors across Membrane Barriers
Source: Sensors (Basel). 2023 Mar 4;23(5):2824. doi: 10.3390/s23052824 (PMC10007193; doi:10.3390/s23052824)
Supplement: Supplementary file 1 [file sensors-23-02824-s001.zip › sensors-2217545-supplementary.pdf]

Supporting information

Optical Penetration of Shape-Controlled Metallic Nanosensors across Membrane Barriers

Ancheng Da [1], Yanan Chu [1], Jacob Krach [1], Yunbo Liu [1], Younggeun Park [2] and Somin Eunice Lee [1,*]

[1] Department of Electrical & Computer Engineering, Biomedical Engineering, Biointerfaces Institute, Applied Physics, Macromolecular Science & Engineering, University of Michigan, Ann Arbor, MI 48109, USA
[2] Department of Mechanical Engineering, University of Michigan, Ann Arbor, MI 48109, USA
* Correspondence: sleee@umich.edu

Supplementary Figures

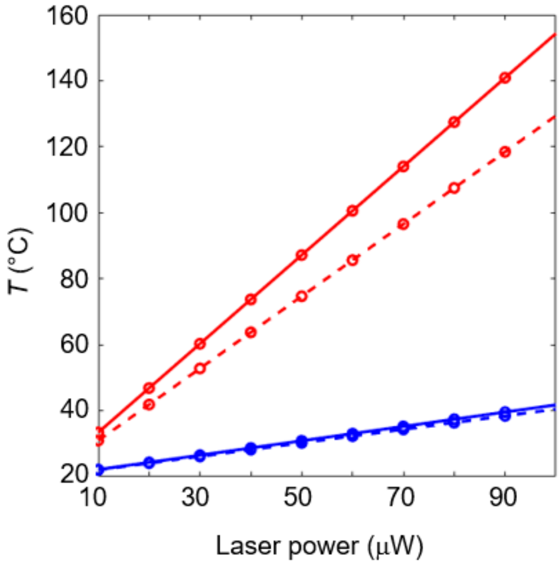

Figure S1. Photothermal heat generation of metallic-based and dielectric-based nanosensors. Interface temperature for AuNR (red solid line), AuBP (red dashed line), siNR (blue solid line), siBP (blue dashed line). Nominal radius r = 50nm, aspect ratio = 4.

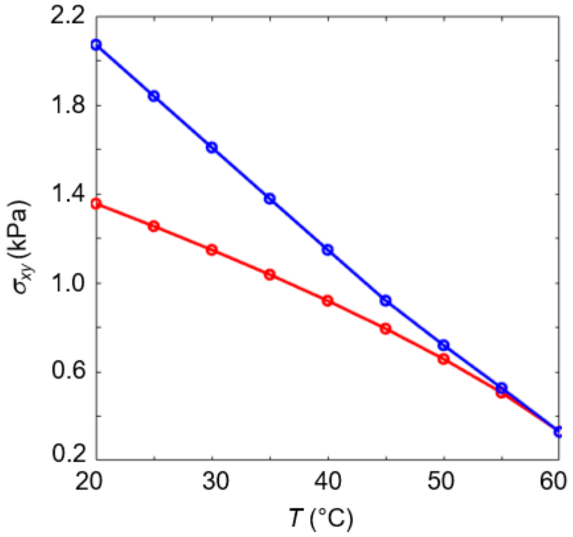

Figure S2. Lateral stress of AuNR and AuBP under environmental temperature variation.

Average interface stress shown for AuBP (blue line), and AuNR (red line).

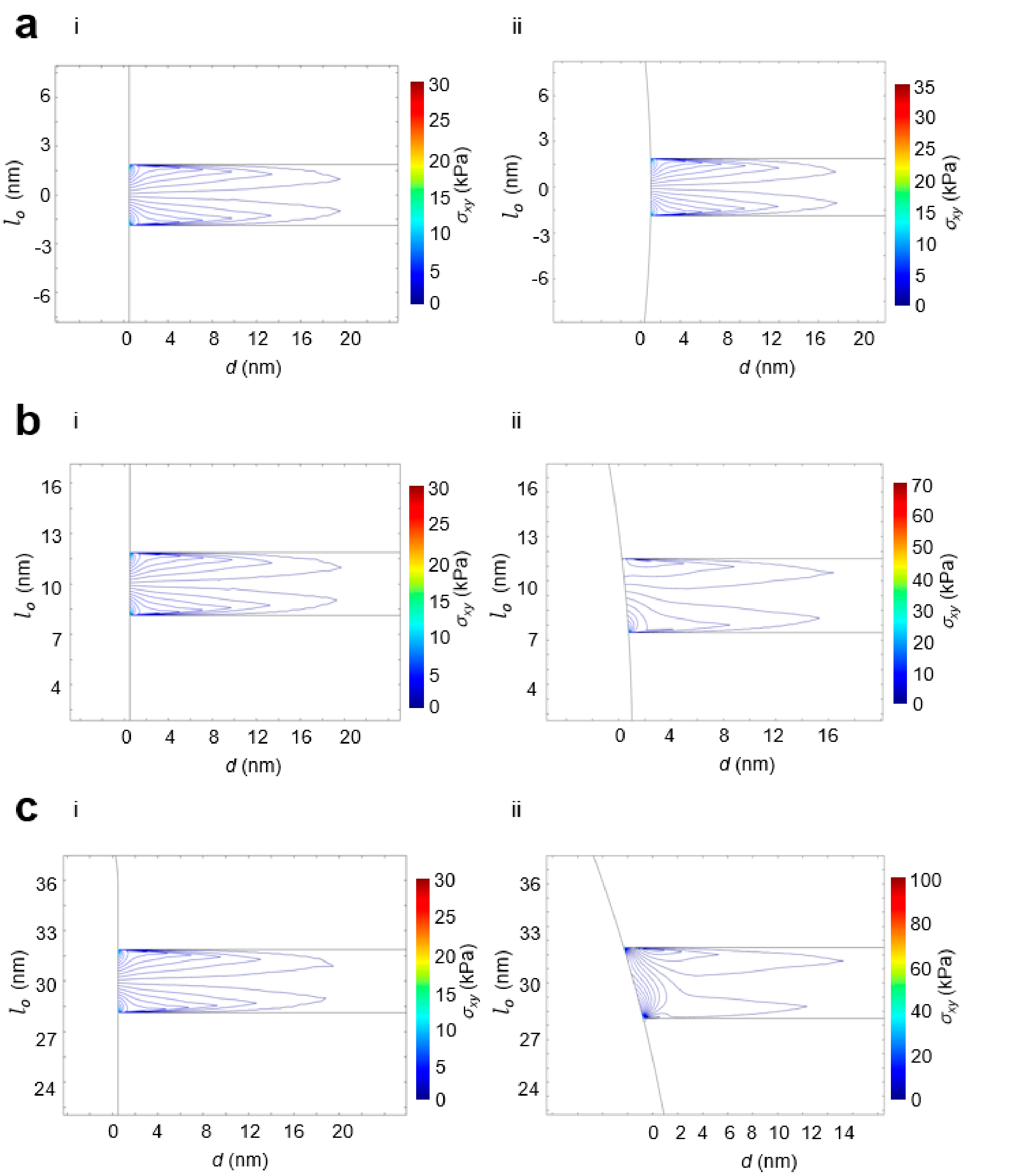

Figure S3. Local lateral stress at the nanoparticle-membrane interface. Local lateral stress contours at the nanoparticle-membrane interface as a function of l_o. **(a) i)** AuNR and **ii)** AuBP. l_o = 0 nm, ω = 10 kHz, **(b) i)** AuNR and **ii)** AuBP. l_o = 10 nm, ω = 10 kHz, **(c) i)** AuNR and **ii)** AuBP. l_o = 30 nm, ω = 10 kHz.

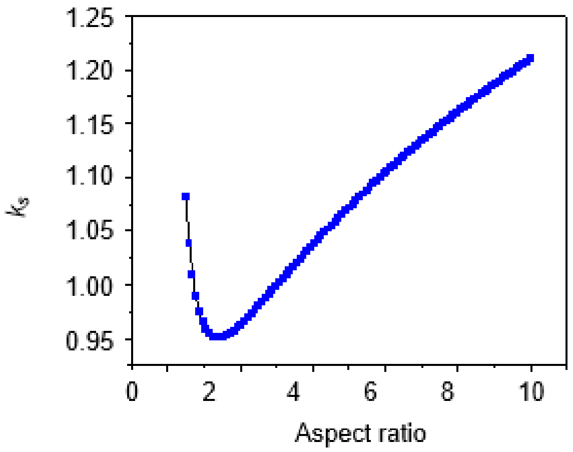

Figure S4. Friction factor. Graph of friction factor k_s versus aspect ratio.
